# Development and validation of LGBTQIA+ search filters: report on process and pilot filter for queer women

**DOI:** 10.5195/jmla.2025.2002

**Published:** 2025-04-18

**Authors:** Hannah M. Schilperoort, Andy Hickner, Jane Morgan-Daniel, Robin M. N. Parker

**Affiliations:** 1 schilper@usc.edu, Head, Wilson Dental Library and Associate University Librarian, USC Libraries, University of Southern California, Los Angeles, CA, United States; 2 alh4014@med.cornell.edu, Education and Outreach Librarian, Samuel J. Wood Library, Weill Cornell Medicine, New York, NY, United States; 3 morgandanie.jane@ufl.edu, Community Engagement & Health Literacy Liaison Librarian, Health Science Center Libraries, University of Florida, Gainesville, FL, United States; 4 robin.parker@dal.ca, Evidence Synthesis Librarian, W.K. Kellogg Health Sciences Library, Dalhousie Libraries, Dalhousie University, Halifax, Nova Scotia, Canada

**Keywords:** LGBTQIA+, lesbians, women who have sex with women, WSW, queer women, bisexual women, search hedge validation, search filter validation, relative recall, systematic reviews as topic

## Abstract

**Introduction:**

A search filter for studies involving lesbian, gay, bisexual, transgender, queer, intersex, asexual, and additional sexual minority and gender identities (LGBTQIA+) populations has been developed and validated; however, the filter contained very small gold standard sets for some populations, and terminology, controlled vocabulary, and database functionality has subsequently evolved. We therefore sought to update and re-test the search filters for these selected subgroups using larger gold standard sets. We report on the development and validation of two versions of a sensitivity-maximizing search filter for queer women, including but not limited to lesbians and women who have sex with women (WSW).

**Methods:**

We developed a PubMed search filter for queer women using the relative recall approach and incorporating input from queer women. We tested different search combinations against the gold standard set; combinations were tested until a search with 100% sensitivity was identified.

**Results:**

We developed and tested variations of the search and now present two versions of the strategy with 99% and 100% sensitivity. The strategies included additional terms to improve sensitivity and proximity searching to improve recall and precision.

**Conclusions:**

The queer women search filters balance sensitivity and precision to facilitate comprehensive retrieval of studies involving queer women. The filters will require ongoing updates to adapt to evolving language and search platform functionalities. Strengths of the study include the involvement of the population of interest at each stage of the project. Future research will include development and testing of search filters for other LGBTQIA+ subgroups such as bisexual and transgender people.

## INTRODUCTION

Locating studies on lesbian, gay, bisexual, transgender, queer, intersex, asexual, and additional sexual minority and gender identities (LGBTQIA+) populations is particularly complex. Researchers may be interested in specific subgroups, such as women who have sex with women (WSW), lesbians, men who have sex with men (MSM), or transgender people, or in combinations of subgroups. Populations may be defined by sexual preference, sexual behavior, how one identifies, or gender identity [1]. Terminology has evolved rapidly over the past several decades and researchers do not always use standard terms to describe the population [2]. Research data on specific subgroups are often buried in articles that use umbrella terms and acronyms such as LGBTQIA+ or sexual orientation and gender identity (SOGI) minorities. These result in searches that retrieve a large number of false positives when trying to identify research data on specific subgroups.

Search filters, also called search hedges, are collections of keywords, variations, and (where available) controlled vocabulary, combined with Boolean operators, that represent concepts in a database search [3]. Filters are useful for exhaustive searches, such as those conducted as part of systematic reviews and other evidence synthesis projects. Filters have been developed to capture a wide range of frequently used concepts, ranging from research methodologies to geography to populations [4]. The relative recall method is a common method of internal search filter validation that involves testing the performance of the filter against a ‘gold standard’ set of database records, defined as “a reference standard against which to establish the performance of the filter” [5]. In the relative recall method, the ‘gold standard’ set of articles is developed by 1) identifying relevant search terms, 2) using those search terms to search for relevant review articles in a database, and 3) screening those review articles to identify a set of original research articles, the ‘gold standard’ set, relevant to the concept [5]. Finally, various search combinations are tested in a database to try to retrieve 100% sensitivity, or 100% recall of the articles in the gold standard set [5].

A PubMed filter for LGBTQIA+ populations has been previously developed by Lee et al. [6] and internally validated by Parker et al. [7] using the relative recall method. Parker et al. [7] concluded that larger gold standard sets for less researched subgroups, such as WSW and bisexual people, would improve validation and performance of the search filter. Furthermore, relevant new Medical Subject Headings (MeSH) were subsequently introduced, including ‘Sexual and Gender Minorities’ in 2018 and ‘Intersex Persons’ in 2020. Several members of the Medical Library Association (MLA) LGBTQIA+ Caucus formed a team to update and re-test the search filters for the underrepresented subgroups using the relative recall internal validation approach and larger gold standard sets with input from the LGBTQIA+ community. Based on subsets with very small development and validation sets from previous work by Parker et al. [7], subgroups of the larger LGBTQIA+ population were prioritized for further development and internal validation of search filters that can be applied in PubMed to comprehensively retrieve relevant records. These groups include: 1) transgender people, 2) bisexual people, 3) queer women (e.g., lesbians, bisexual women, women who have sex with women, etc.), 4) intersex people, and 5) asexual people. In this article, we will focus on one example subgroup, queer women, which we used to pilot our process. The purpose was to develop a sensitivity-maximizing search filter [8] that would retrieve more relevant articles on queer women.

## Methods

### Action Plan and Protocol

This article focuses on the development and validation of a search filter for queer women and is written by the four authors who conducted this subset validation project. The queer women search filter validation project is part of the larger LGBTQIA+ search filter project initiated by a larger group of researchers from the Medical Library Association (MLA) LGBTQIA+ Caucus. This larger team created an action plan and research protocol to coordinate the work of the larger project. The LGBTQIA+ search filter action plan was adapted from the action plan developed for the MLA Latinx Caucus and their Hispanic/Latinx Inclusive Terminologies Project [9]. Our action plan covered logistical issues such as project and team management, tools for collaboration, and goals for dissemination. The protocol outlines the research objectives and approach, as described in the rest of this methods section. We did not conceive of the protocol as a strict guideline for the methods, but as a living document that we modified through conducting this pilot with a single subgroup. See the link in our Data Availability Statement to view our action plan and protocol.

### Defining Subgroups

Definitions are crucial in research involving LGBTQIA+ populations where there is a need to balance precision with inclusion. Terms such as LGBTQIA+ and SOGI collate together populations distinguished by sexual orientation and gender identity. Sexual orientation is in turn assessed along the dimensions of attraction, behavior, and identity [1]. For our pilot with the queer women filter, our definition encompassed both sexual behavior (women who have sex with women) and identity (women who identify as lesbian, bisexual, or queer). We defined ‘women’ based on how the author of the included study described the population, rather than on assignment as female at birth (AFAB). For example, articles describing transgender women who have sex with women were included, whereas articles focusing specifically on nonbinary or transgender AFAB people who did not identify as women were not. We intentionally used the phrase ‘queer women’ for our filter and throughout this article to describe this population and encompass the broad spectrum of women whose sexual orientation is not exclusively heterosexual. Although the term ‘queer’ has been used as a slur against LGBTQIA+ people in the past, today it has been reclaimed by many in the LGBTQIA+ community as an inclusive term that includes the broad spectrum of gender and sexual orientation identities within the community [10]. We define ‘queer women’ as women who identify as lesbian, bisexual, pansexual, queer, not exclusively heterosexual, or who have (or have had) sex with women.

### Creation of the Gold Standard Set

We searched PubMed using the WSW filter from Parker et al. [7] along with additional terms brainstormed by the current team. The population search concept was combined with a search filter for systematic or scoping reviews using a strategy developed by Salvador-Oliván et al. [11] to retrieve research review publication types; no date limit was applied. The previous study Parker et al. [7] worked from a set of only 39 articles for the WSW filter. We aimed to develop a test set of at least 200 records, which is double the 100 records Sampson et al. [5] suggested for internal validation. To ensure that the filter applies to a variety of topics, we specified that references from a minimum of five reviews would be used to develop the gold standard set.

To identify original research articles for the development of the queer women gold standard set, potentially relevant reviews were screened by two team members using the following eligibility criteria. Reviews had to focus specifically on WSW or queer women populations or more broadly on the larger LGBTQIA+ population. We included systematic reviews, scoping reviews, narrative reviews, and other types of evidence-synthesis and secondary literature related to the target population. To meet the inclusion criteria, at least one study included in the review must have focused on queer women and the review must separately report data related to queer women. We selected a purposive sample of reviews for reference checking to ensure a breadth of topics and domains (e.g., psychology/social work, biomedical, clinical medicine) and demographics (e.g., youth, geriatric, adult). Reviews were sorted by ‘most relevant’ in Covidence and reviewed for selection in that order. This sorting feature in Covidence review management software uses machine learning to prioritize records similar to those selected for further review or inclusion after at least twenty-five have been screened [12].

We conducted reference checking for the selected reviews using Scopus on January 11 and February 7, 2023, and exported them to Covidence. We independently screened retrieved records in duplicate first by title/abstract, and then by full text. Because not all records were indexed in MEDLINE, we periodically searched MEDLINE for the records marked for inclusion until we reached just over 200 studies.

During screening, we continued to refine the eligibility criteria. Included studies could report on multiple LGBTQIA+ sub-populations if they presented separate data for queer women. We included studies where data on queer women was only available at full text level. We excluded studies that did not contain separate data for queer women. We also excluded the following types of studies unless it was apparent from title/abstract that the article was about queer women specifically:

- Studies that measured attitudes (e.g., heterosexual/general population attitudes toward/knowledge of homosexuality, same-sex marriage, etc.). This was done to restrict the gold standard set to only original research studies on the health of queer women rather than attitudes toward queer women as well as other members of LGBTQIA+ populations.
- Non-human subjects research (e.g., theoretical models/frameworks, narrative reviews, systematic reviews, or policy position papers that did not contain data from original research). However, we included meta-analyses that reported data specific to queer women.
- Qualitative studies with mixed populations, as it is often difficult to ascertain which data are associated with the queer women participants in these studies.
- Studies published in languages other than English.

### Development of Search Strategies

The research team used several sources to develop a set of terms to search PubMed to identify review articles for the creation of the gold standard set. Sources included terms from Parker et al. [7], personal lived experience, LGBTQIA+ glossaries, mining relevant articles identified through PubMed searches, and the synonyms and alternative terms listed in relevant MeSH and Emtree records. The list of search terms was developed in September and October 2022 and included the following index terms: “Sexual and Gender Minorities”[Mesh:noexp], “homosexuality, female“[Mesh], ‘women who have sex with women’/exp, ‘homosexual female’/exp, and ‘bisexual female’/exp. To develop the final search filter, the team reviewed articles in the gold standard set to identify additional search terms.

### Relative Recall Validation

**Figure 1:**
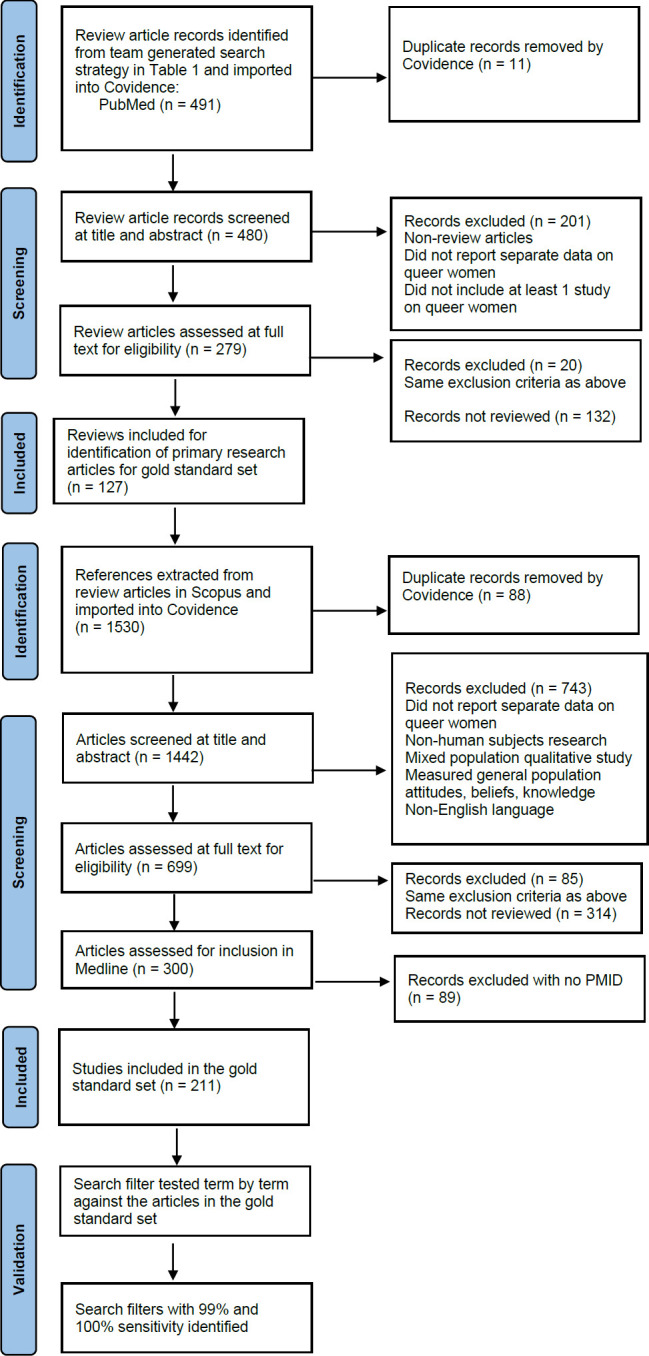
Flow diagram for validation of queer women search filter

Figure 1 is a flow diagram representing the overall internal validation process for the two versions of the queer women search filter developed in this project. We used the gold standard set to internally validate the search filter using the relative recall method of validation described by Sampson et al. [5]. Each term was searched in PubMed and tested against the gold standard set to identify terms contributing to retrieval of articles in the gold standard set. Relative recall was calculated using total gold standard articles retrieved by the search (on the day of testing) divided by the total number of articles in the gold standard set. The resulting number was then multiplied by 100 to calculate the sensitivity of the search as a percentage. Combinations of terms were tested until a search with 100% sensitivity was identified. A search with 99% was also identified to provide an option for a more precise version of a sensitive search.

### Involvement of Queer Women

Three out of four of the authors on the queer women filter validation team identify as queer women and their personal lived experiences were considered when developing the study action plan, search terms and strategies, and the gold standard set. Multiple other members of the larger LGBTQIA+ filter validation team who provided input also identify as queer women. This was done intentionally to ensure that members of the population of interest were included at every stage of the project. We also shared our draft search filters and methodologies through our Medical Library Association (MLA) and Canadian Health Libraries Association (CHLA) networks to solicit additional feedback from queer women librarians and informationists.

## RESULTS

### Gold Standard Set

The initial set of reviews for the creation of the gold standard set were identified by searching PubMed in October 2022 using the terms listed in Table 1 from Parker et al. [7] and terms identified to be relevant by the current research team.

**Table 1:** Search terms for generating the review set

Term source	Terms
Parker et al. ^*^ [7]	“homosexuality, female"[Mesh] OR lesbian^*^[tiab]
Team generated ^**^	“Sexual and Gender Minorities” [Mesh:NoExp] OR “homosexuality, female”[Mesh] OR lesbian^*^[tw] OR “women who have sex with women”[tw] OR WSW[tw] OR “female homosexual^*^”[tw] OR “homosexual female^*^”[tw] OR “homosexual women”[tw] OR “homosexual woman”[tw] OR Lesbigay[tw] OR “women loving women”[tw] OR “gay women”[tw] OR “queer women”[tw] OR “queer woman”[tw] OR “bisexual women”[tw] OR “bisexual woman”[tw] OR “bisexual female^*^”[tw] OR “pansexual women”[tw] OR “sexual minority women”[tw] OR lesbophobi^*^[tw]

* “Sexual and Gender Minorities” [Mesh:NoExp] added after 2018 as noted in the limitations section of Parker et al. [7].

** The team generated terms were combined with the Salvador-Oliván et al. [11] systematic/scoping review filter as described in the “Creation of the Gold Standard Set” section. The Salvador-Oliván et al. [11] can be found in the original publication and in the Action Plan and Protocol for this project linked in our Data Availability Statement.

The team-generated search in Table 1 combined with the Salvador-Oliván et al. [11] systematic and scoping review filter resulted in 491 records of review articles, eleven of which were duplicates identified by Covidence. 480 records were screened within Covidence; each record was screened independently by two team members at title and abstract and full text levels. This selection process identified 127 reviews related to queer women, the references of which were extracted using Scopus. We screened the references obtained from Scopus to verify their relevance to queer women. The title and abstract and full text screening of this set formed the gold standard set for developing the search filter; each record was screened by two team members. Screening continued until the target number of 200 relevant records had been met or exceeded, as described in the methods section. Of the 1,530 references from the reviews imported into Covidence, 88 were identified as duplicates. One thousand four hundred and forty-two were screened at title and abstract, with 743 excluded as irrelevant and 314 not reviewed. Records were screened at full text independently in duplicate until we reached 300 relevant records. Of the 300 records, 211 had PMIDs, producing a PubMed gold standard set that was used to develop the search filters.

### Search Strategies

The relative recall (sensitivity) of each term from the initial set from Table 1 is reported in Table 2. Table 2 also shows the total recall from PubMed for each term on the dates of searching, April 26 and 27, 2023. As a limited function of proximity searching was introduced for PubMed in November 2022 [13], phrases were searched using up to three words between the quoted terms.

**Table 2:** Performance of search terms against PubMed gold standard set

Terms to test	Total records retrieved in PubMed (A) (date: 4/26-4/2/723)	Total gold standard articles retrieved (B)	Sensitivity % (B/total number of articles in gold standard development set X 100)
“Sexual and Gender Minorities”[Mesh:NoExp]	10,229	**39**	**18.5**
“homosexuality, female”[Mesh]	4,192	117	**55.5**
lesbian^*^[tw]	8,805	181	**85.8**
lesbian^*^[tiab]	8,804	181	**85.8**
“Lesbian women”[tw]	600	24	**11.4**
“Lesbian woman”[tw]	21	1	**0.5**
lesbians[tw]	1,929	68	**32.2**
“women who have sex with women”[tw]	205	3	**1.4**
WSW[tw]	200	1	**0.5**
“female homosexual^*^”[tw]	177	2	**0.9**
“homosexual female^*^”[tw]	31	1	**0.5**
“female homosexual”[Title/Abstract:~3]	154	1	**0.5**
“homosexual women”[tw]	111	2	**0.9**
“homosexual women”[Title/Abstract:~3]	347	6	**2.8**
“homosexual woman”[tw]	7	0	**0.0**
“homosexual woman”[Title/Abstract:~3]	20	0	**0.0**
Lesbigay[tw]	5	0	**0.0**
“women loving women”[tw]	9	0	**0.0**
“gay women” [tw]	27	0	**0.0**
“queer women” [tw]	80	2	**0.9**
“queer woman” [tw]	7	0	**0.0**
“queer women” [Ti tie/Abs trac t:~3]	141	3	**1.4**
“queer woman” [Title/Abstract:~3]	11	0	**0.0**
“bisexual women” [tw]	725	47	**22.3**
“bisexual woman” [tw]	12	0	**0.0**
“bisexual women” [Ti tie/Abs trac t:~3]	1,079	57	**27.0**
“bisexual woman” [Title/Abstract:~3]	21	0	**0.0**
bisexual female^*^ [tw]	110	4	**1.9**
“bisexual female” [Title/Abstract:~3]	198	1	**0.5**
“bisexual females” [Title/Abstract:~3]	148	5	**2.4**
“pansexual women” [tw]	5	0	**0.0**
“sexual minority women” [tw]	576	35	**16.6**
lesbophobi^*^[tw]	8	0	**0.0**
sexual minority female^*^ [tw]	59	1	**0.5**
sexual minority women[tw]	576	35	**16.6**
“sexual minority females” [Title/Abstract:~3]	68	2	**0.9**
“sexual minority female” [Title/Abstract:~3]	99	3	**1.4**
“sexual minority women” [Title/Abstract:~3]	729	40	**19.0**
“sexual minority woman” [Title/Abstract:~3]	7	0	**0.0**
“nonheterosexual women” [Title/Abstract:~3]	23	3	**1.4**
“same sex women” [Title/Abstract:~3]	307	4	**1.9**

We conducted iterative testing of the terms in Table 1 through combinations based on retrieval of at least one record in the PubMed gold standard set. Further testing of combinations was completed based on examination of the records not retrieved by the baseline search from Parker et al. [7] and adding phrases with proximity operators and truncation to the base search. We used the combination field search [TW] on individual terms for increased sensitivity, but at the time of the search, PubMed's proximity search only permitted use of the slightly more focused combination field search [TIAB]. Similarly, at the time of testing searches, proximity searching in PubMed did not permit use of truncation on any of the terms within the phrase.

Table 3 shows the performance of the various search combinations, along with the Most Sensitive and Optimized Sensitive Search strategies that retrieved 100% (Table 4) and 99% (Table 5) of the PubMed gold standard set, respectively. The additional terms that improved the retrieval of the Most Sensitive Search filter are highlighted in bold. The Optimized Sensitive Search substantially reduced the Number Needed to Read (NNR) [14] compared to the Most Sensitive search when calculated from the gold standard set and PubMed retrieval numbers on May 15, 2023 (58.39 vs 81.69). NNR represents search precision and is calculated for each search strategy with the following formula: NNR = Total # records retrieved/# GS records retrieved.

**Table 3:** Performance of select search combinations

Test search (5/15/2023)	Total records retrieved in Pubmed	Gold standard articles retrieved (out of 211)	Sensitivity %	Number Needed to Read
“homosexuality, female” [Mesh] OR lesbian* [tiab] from Parker et al. [7]	9850	191	90.52%	51.57
Strategy used to get reviews for gold standard (Table 1)	17862	201	95.26%	88.87
Optimized Sensitive Search (Table 5)	12203	209	99.05%	58.39
Most Sensitive Search (Table 4)	17236	211	100.00%	81.69

**Table 4:** Most sensitive search

“homosexuality, female” [Mesh] OR lesbian*[tw] OR “sexual minority females” [tiab:~3] OR “sexual minority female” [tiab:~3] OR “sexual minority women” [tiab:~3] OR “bisexual women” [tiab:~3] OR “bisexual female” [tiab:~3] OR “bisexual females”[tiab:~3] OR “homosexual women” [tiab:~3] OR “female homosexual”[tiab:~3] OR ((“women” [tw] OR “female”[tw]) AND (“sexual minorit*”[tw] OR “non-heterosexual*” [tw] OR nonheterosexual*[tw] OR **“same sex”[tw])) OR ((“women”[tw] OR “female”[tw]) AND (“same gender”[tiab:~3] OR “same sex”[tiab:~3]) AND “attracted”[tiab])**

**Table 5:** Optimized sensitive search

(("homosexuality, female” [Mesh] OR lesbian*[tw] OR “sexual minority females”[Title/Abstract:~3] OR “sexual minority female” [Title/Abstract:~3] OR “sexual minority women”[Title/Abstract:~3] OR “bisexual women”[Title/Abstract:~3] OR “bisexual female” [Title/Abstract:~3] OR “bisexual females” [Title/Abstract:~3] OR “homosexual women”[Title/Abstract:~3]) OR ((“women”[tw] OR “female”[tw]) AND (sexual minorit*[tw] OR non-heterosexual* [tw] OR nonheterosexual*[tw]))) or “female homosexual” [Title/Abstract:~3]

For full data on testing of the filters and each of the various terms, as well as the search strategy for the PubMed gold standard set, please refer to the Data Availability Statement at the end of this article.

## DISCUSSION

One of the strengths of our methodology was the inclusion of queer women on the study team to center the knowledge and experience of people in the study population. Including people with lived experience, borrowed from Weeks and Hoskins [15], helped the team to consider current and emerging language used by people in the target study population that may not be commonly found in the research literature. Because the language used in research literature is often mismatched with everyday language used by populations, not all the emerging terminology considered by the team made it into the final search filter, as it was not needed to capture the published research related to this population. However, because language is constantly evolving, knowledge of emerging terminology is important. Many terms used by the population may eventually make it into the published research literature, so keeping up to date with emerging terminology is important for future updates of the search filter.

Another strength of our methodology was the incorporation of feedback from other health sciences librarians outside of the study team. We presented our preliminary findings at the Canadian Health Libraries Association (CHLA) meeting in Halifax, Nova Scotia in June 2023 [16]. We shared a Google folder with our protocol, search strategies, and search testing. We asked participants to provide feedback on any aspect of our study, including the protocol and development of the gold standard set and search strategies and terms. After the meeting, we shared the same Google folder with the MLA LGBTQIA+ Caucus for further feedback, especially from those who identify as queer women. We did not receive much feedback, but feedback we did receive included helpful suggestions for new and emerging terms relevant to the queer women population, but these terms have not yet made it to the published research literature and therefore were not included in the search filters.

The two versions of our queer women search filter built and expanded upon previous filters [6, 7]. As such, we updated the filters to consider additional MeSH and keywords based on a larger gold standard validation set. We expanded the gold standard set by including articles on the broader LGBTQIA+ population in which data were reported separately for queer women, in addition to articles that focused specifically on queer women. In alignment with the Parker et al. [7] findings, we found that data pertaining specifically to queer women are often buried in studies about the broader LGBTQIA+ population, making it challenging for researchers to find data on this subpopulation. This is demonstrated in this validation study by the significant decrease in precision necessary to achieve 100% retrieval of the gold standard set with the Most Sensitive Search, compared to the Optimized Sensitive Search. In addition to the paucity of research that specifically targets queer women, we observed that most of the studies that are specific to queer women were psychosocial rather than biomedical. These findings highlight the need for further biomedical, as well as general health, research that is specific to queer women.

Because data regarding queer women are so often embedded within studies for the broader LGBTQIA+ population, our Most Sensitive queer women search filter had to be broad to capture all the relevant data. This means that the search filter, in addition to retrieving data on queer women, will retrieve articles on the broader LGBTQIA+ population as well. Researchers who use either of the two versions of this search filter will have to spend more time and effort to extract the relevant data for queer women from these articles, but the time and effort spent will result in more comprehensive data and allow for more informed conclusions and recommendations. Both versions of the search filter represent a balance of sensitivity, to capture queer women data embedded in larger LGBTQIA+ studies, and precision, to specifically target studies concerning queer women. We have also reported the second most sensitive search, the Optimized Sensitive Search, as an option to improve precision and further reduce the number needed to read to identify relevant studies. When used for evidence synthesis research, population search filters are combined with search strategies for one or more additional concepts, such as intervention, exposure, or context. Therefore, selecting the Most Sensitive or Optimized Sensitive filter will depend on the purpose of the search and the degree of precision and sensitivity that can be achieved in the strategies for the other concepts. For example, for topics with very little available evidence, such as some biomedical concerns, researchers may opt for the Most Sensitive Search filter to improve retrieval of any relevant studies. On the other hand, for social phenomena that are harder to search precisely, such as social determinants of health [17], the use of the Optimized Sensitive Search would contribute to feasibility by decreasing the number of records needed to screen while remaining robust for sensitivity.

The Most Sensitive search that captured all articles in our gold standard set included less common search terms such as ‘same sex’ and ‘same gender’ that were used in articles published in geographical areas outside of North America. As using these search terms reduced precision, we combined them with gender-specific terms to retrieve phrases such as “women attracted to the same sex” but not “men attracted to the same sex.” Though these variations add complexity to the search filter, they improve recall and precision and were found to be important for capturing studies that include bisexual and nonheterosexual women. This resulted in a more comprehensive data set which captured the nuances of the wider population of queer women. As language evolves, the search filters will have to be updated with new and emerging terminology. For example, constructive feedback from the community offered terms not included in the search filters, such as ‘sapphic’ or ‘omnisexual’, which were not used by any articles in our gold standard set. Other phrases such as “women who have sex with women” are not included in the final iteration of the filters because they did not improve sensitivity within the gold standard set. “Sexual and Gender Minorities”[MeSH] was not included in the final iteration of the filters because it incorporates the entire LGBTQIA+ community and would dramatically decrease precision and increase NNR. Though these terms were not necessary to capture 100% of the articles relevant to queer women in the gold standard set, future research could explore external validation using both emerging and inclusive terms such as these.

Changes to the PubMed search platform influence search retrieval in both positive and negative ways. Proximity searching functionality was added to PubMed in November 2022 [13]. This allowed us to incorporate additional terms while also maintaining more precision than would be possible without proximity. On the other hand, fully automated MeSH indexing, which was implemented in April 2022 [18], may result in decreased search retrieval precision or sensitivity going forward if relevant articles are indexed incorrectly or with the broader and related MeSH terms (e.g., “Sexual and Gender Minorities”[Mesh] or “Homosexuality”[Mesh]) [19]. It is inevitable that search platforms such as PubMed will continue to change and evolve, which will influence search retrieval in various ways. As the platform evolves, the search filter will have to be updated to adjust to new functionalities.

## LIMITATIONS

As previously mentioned, a limitation of the search filter is the need to include a wide range of search terms to capture data on queer women that are often buried in full text of studies on the broader LGBTQIA+ population. This says more about the paucity of research, especially biomedical research, that specifically focuses on queer women's health than the validity of the search filter.

A limitation of our methodology is that we could have developed a gold standard set of larger than 200. As previously mentioned, we settled on a gold standard set of 200, which is twice the number of a minimum gold standard set recommended by Sampson et al. [5] and significantly more than the thirty-nine article gold standard set in Parker et al. [7]. A larger target set may have enabled us to identify records using a few additional less common and emerging terms, resulting in development of an even more comprehensive search filter. We chose to stop at 200 to balance time and effort spent, in recognition of the impact on search filter stability of the evolving nature of language and social constructs such as sexual orientation and identity.

A related limitation concerns the use of relative recall to create the gold standard set for internal validation, as pulling references from published reviews is inherently retrospective. We will address this limitation for the future stages of the project by also screening records that have cited the reviews from which we will pull references, creating a second gold standard set to use for external validation of the search strategies developed for the other subgroups.

We used PubMed to search MEDLINE because it is the largest and most used free biomedical and health sciences search platform. A limitation of using PubMed is that the PubMed phrase index does not include some phrases used in our search filter, which may result in additional terms contributing to retrieval on other search platforms, such as Ovid MEDLINE. This impact may have been partially mitigated using PubMed's recently added proximity search feature. However, at the time of the search, PubMed's proximity search could only be done using the [Title], [Title/Abstract], or [Affiliation] fields. Thus, for phrases that required the precision of proximity searching, we used the [Title/Abstract] instead of the [Text Word] tag, which may have resulted in a loss of articles that included these phrases that may have been included in the additional [Text Word] fields not included in the [Title/Abstract] fields.

Another limitation is that our search filters are designed for use in evidence synthesis projects, where records are generally screened by title and abstract as opposed to affiliation or journal title. Finding literature based on the affiliation or journal title fields is outside the scope of our project.

### Directions for Future Research

These queer women search filters are part of a larger project to update and re-validate previously validated PubMed search filters for LGBTQIA+ populations. The goal of our larger project is to consider the impact of new MeSH terms and keywords on the relative recall of particular subpopulations of the LGBTQIA+ community. In addition to the queer women filters, our goal is to also develop larger gold standard sets for bisexual, transgender and nonbinary, intersex, and asexual populations. In addition to the creation of the subpopulation filters, new MeSH terms and keywords we identify will also be incorporated into a larger LGBTQIA+ search filter to capture research pertaining to the broader population.

A future phase of this project will involve eliciting feedback on each subgroup filter from members of that community. We will use this feedback to develop a larger list of relevant terms to describe each subgroup (and relevant research related to the population in question), recognizing that not all terms may be currently used in health-related literature and acknowledging that harmful language may have been used historically [20]. The resulting search filters will be validated using the relative recall method to create gold standard sets, with more recent records identified by screening the citations of articles citing the included reviews.

## CONCLUSION

Population search filters provide a helpful starting place for researchers conducting evidence synthesis. However, because language is constantly evolving, published population search filters can never represent all relevant search terms and emerging language. The search filters will need to be revisited on an ongoing basis to account for the continual evolution of language. They also need to be adapted for the needs of each evidence synthesis project and its use in research. For example, the Most Sensitive Search for queer women uses a complex PubMed search strategy with proximity operators for maximum retrieval, such as would be used in large systematic or scoping reviews in combination with searches for one or more well-defined concepts. The Optimized Sensitive Search also has very high sensitivity and uses proximity functions to retrieve relevant records with a reduced screening burden, useful for rapid reviews or for topics that are hard to define or have a high volume of search results. The search filters for queer women reported here serve as an evidence-based starting point for anyone seeking health research for this understudied population.

## Data Availability

Data associated with this article are available in the Open Science Framework at https://osf.io/brxwt.

## References

[1] Sell R, Conron K. Definitions: ‘Straight, that is not gay’: Moving beyond binary notions of sexual and gender identities. In: Stall R, Dodge B, Bauermeister JA, Poteat T, Beyrer C, eds. LGBTQ health research: Theory, methods, practice. Baltimore, MD: Johns Hopkins University Press; 2020. 61–90.

[2] Eliason MJ. An exploration of terminology related to sexuality and gender: Arguments for standardizing the language. 2014. 29(2):162–175. 10.1080/19371918.2013.775887. 24405201

[3] White VJ, Glanville JM, Lefebvre C, Sheldon TA. A statistical approach to designing search filters to find systematic reviews: Objectivity enhances accuracy. J Inf Sci. 2001. 27(6):357–370. 10.1177/016555150102700601.

[4] Jenkins M. Evaluation of methodological search filters - A review. Health Info Libr J. 2004. 21(3):148–163. 10.1111/j.1471-1842.2004.00511.x. 15318913

[5] Sampson M, Zhang L, Morrison A, Barrowman NJ, Clifford TJ, Platt RW, Klassen TP, Moher D. An alternative to the hand searching gold standard: validating methodological search filters using relative recall. BMC Med Res Methodol. 2006. 6(33). 10.1186/1471-2288-6-33. PMC1557524 16848895

[6] Lee JGL, Ylioja T, Lackey M. Identifying lesbian, gay, bisexual, and transgender search terminology: A systematic review of health systematic reviews. PloS one. 2016. 11(5):e0156210. 10.1371/journal.pone.0156210. 27219460 PMC4878791

[7] Parker RMN, Wanner A, Foster M, Lackey M. Design & Validation of Search Filters for LBGTQ+ Populations. Cochrane Colloquium Edinburgh. September 17, 2018, Edinburgh, Scotland. https://colloquium2018.cochrane.org/abstracts/design-and-validation-search-filter-lgbtq-populations.

[8] Glanville J, Bayliss S, Booth A, Dundar Y, Fernandes H, Fleeman ND, Foster L, Fraser C, Fry-Smith A, Golder S, Lefebvre C, Miller C, Paisley S, Payne L, Price A, Welch K. So many filters, so little time: The development of a Search Filter Appraisal Checklist. Journal of the Medical Library Association. 2008; 96(4): 356–61. 18974813 10.3163/1536-5050.96.4.011 PMC2568852

[9] Weeks A, Nugent R, Orozco R, Roth S, Fell S, Hoskins K, Shields T, Abad E, Vidales J, Corn M, Hanneke R, Lackey M, Lokker C, Navarro-Ruan T, Ramirez M, Scheinfeld L, Burns H. Hispanic/Latinx Inclusive Terminologies Projects Action Plan [Internet]. 2021. [cited 12 Apr 2024]. https://osf.io/6myqs.

[10] HRC Foundation. Glossary of Terms [Internet]. Human Rights Campaign. [cited 21 Oct 2024]. https://www.hrc.org/resources/glossary-of-terms.

[11] Salvador-Oliván JA, Marco-Cuenca G, Arquero-Avilés R. Development of an efficient search filter to retrieve systematic reviews from PubMed. J Med Libr Assoc. 2021. 109(4):561–574. 10.5195/jmla.2021.1223. 34858085 PMC8608217

[12] Veritas Health Innovation. Covidence Systematic Review Software [Internet]. 2024. [cited 12 Apr 2024]. www.covidence.org.

[13] National Library of Medicine. PubMed update: Proximity search now available in PubMed. NLM Tech Bull. 2022. Nov-Dec(449): e4. https://www.nlm.nih.gov/pubs/techbull/nd22/nd22_pubmed_proximity_search_available.html.

[14] Bachmann LM, Coray R, Estermann P, Ter Riet G. Identifying diagnostic studies in MEDLINE: reducing the number needed to read. J Am Med Inform Assoc. 2002. 9(6):653–658. doi:10.1197/jamia.m1124. 12386115 PMC349381

[15] Weeks A, Hoskins K. Hispanic/Latinx Inclusive Terminologies Projects Technical Report [Internet]. 2022. [cited 12 Apr 2024]. https://digitalscholarship.unlv.edu/lib_articles/735.

[16] Schilperoort H, Hickner A, Parker R, Foster M, Misquith C, Morgan-Daniel J, Saylor K, Shields T, Toole F. Update and Re-Validation of Search Filters for LGBTQIA+ Populations [Internet]. Open Science Framework. 2023. https://osf.io/fnkq2.

[17] Hanneke R, Brunskill A. Searching for the social determinants of health: observations from evidence synthesis publications. *Syst Rev* 13, 134 (2024). 38755700 10.1186/s13643-024-02551-y PMC11097542

[18] National Library of Medicine. Automated indexing FAQs [Internet]. 2024. [cited 9 Oct 2024]. https://support.nlm.nih.gov/kbArticle/?pn=KA-05326

[19] Chen E, Bullard J, Giustini D. Automated indexing using NLM's Medical Text Indexer (MTI) compared to human indexing in Medline: A pilot study. J Med Libr Assoc. 2023. 111(3): 684–694. 10.5195/jmla.2023.1588. 37483360 PMC10361558

[20] Taubman Library. Language Matters: Handling Tough Terms in Systematic Searching [Internet]. 2024. [cited 12 Apr 2024]. https://guides.lib.umich.edu/TaubmanTalks/ToughTerms.

